# The impact of soil acidification on cementing substances and aggregate stability

**DOI:** 10.1371/journal.pone.0318417

**Published:** 2025-04-16

**Authors:** Xiaoxu Zhu, Shengchen Zhao, Siqi Lin, Jihong Wang, Su Leng

**Affiliations:** College of Resource and Environmental Science, Jilin Agricultural University, Changchun, Jilin, China; ICAR - IIFSR: ICAR - Indian Institute of Farming Systems Research, INDIA

## Abstract

The excessive utilization of chemical fertilizers, particularly nitrogen fertilizers, is leading to decline in the pH level of the black soil in Jilin Province. Acidification of black soil leads to reduced salt base saturation, decreased organic matter content, and increased soil degradation, which, in turn, leads to diminshed aggregate stability and poor soil structure, negatively affecting soil fertility. As a result, the sustainability of food production and farmland ecosystem stability are at risk. The precise relationship between alterations in cementing substances and changes in soil aggregate stability during the acidification of black soil remains unclear, and the ultrasonic thermal difference method allows for the quantitative description of changes in soil aggregate stability. Therefore, this study employed the ultrasonic thermal difference method to investigate the impact of acidification on the stability of black soil aggregates and their cementing substances through a simulated fertilizer drenching experiment, thus elucidate the relationship between primary cementing materials and the stability of aggregates under varying degrees of black soil acidification, and to provides theoretical basis and data for alleviating and preventing acidification of black soil in Jilin Province. The results disclosed a gradual decline in soil organic carbon (SOC) levels during the acidification experiment, while water-soluble organic carbon (WSOC) first increased and then decreased. After 25 years of simulated leaching, SOC decreased by 1.34% and WSOC declined by 15.63%. Acidification has a minimal impact on Fe-Al bonded organic carbon but significantly reduces calcium-bonded organic carbon by 17.07% over 25 years. The content of exchangeable Ca^2+^ and Mg²⁺ decreases as acidification intensifies. After 25 years, exchangeable Ca^2+^ and Mg²⁺ decreased by 9.42% and 7.00%, respectively. The acidification of the test soil resulted in a 46.5% reduction in the aggregate stability energy (E) of water-stable microaggregates, with an average decrease of 14.04 J/g for every 0.1 unit decrease in pH. Additionally, the soil critical stabilization energy (Ecrit) exhibited a 51.48% reduction. The results demonstrated that a decrease of 0.32 J/g in E was associated with a 0.1 unit decrease in pH on average. Furthermore, the multivariate linear regression analysis revealed that the reduction in soil organic carbon (SOC) content contributed the most to the decline in E, followed by Calcium bond-bound soil organic carbon (Ca-SOC). Notably, Ca-SOC exerted the greatest influence on the reduction in sand grain Ecrit, followed by SOC.

## 1. Introduction

Soil acidification is a biogeochemical process that occurs simultaneously with soil formation and evolution. Under natural conditions, it can take decades or centuries to become discernible [1]. However, increased industrial and agricultural activities have significantly augmented the emission of SO₂, NO_X, and NH₃, thereby accelerating soil acidification [2,3]. Literature review reveals that global terrestrial nitrogen deposition exceeded 50 kg/ha from 2000 to 201 [4]. The Calhoun Forest Experiment Station in South Carolina revealed that natural and human-generated acidogenic substances transformed the chemical composition of soil exchangeable complexes. pH declined by one unit in the 0–15 cm layer, by 0.4 units in the 15–35 cm layer, and by 0.3 units in the 35–60 cm layer, with an average cation loss of 1.57 kmol/ha/yr in the 0–60 cm layer. The active and total acids increased by averages of 1.26 and 3.28 kmol/ha/yr, respectively [5]. A study by Watmough et al. [6] examined the inputs and outputs of sulfate, nitrate nitrogen, ammoniacal nitrogen, calcium, magnesium, and potassium in 21 forested catchments across 17 regions in Canada, the United States, and Europe. Despite declining sulfate deposition, it still remains a major contributor to soil acidification in many regions. Acid deposition is frequently accompanied by alkali deposition, which serves to alleviate or even counteract the effects of acid deposition on soil acidification. The excessive use of chemical fertilizers, particularly chemical nitrogen fertilizers, constitutes the primary controlling factor in the global acidification of agricultural soils [7]. An extensive study performed in south-central Wisconsin indicated that the application of ammonia nitrogen fertilizer brought about a significant increase in exchangeable acids, accompanied by a reduction in cation exchange, salt base saturation, and the content of exchangeable calcium and magnesium [8]. About 19% of the UK’s arable land is acidified [9]. In Crossfield, Alberta, a 27-year soil trial involving ammonium nitrate fertilizer evinced a conspicuous decline in soil pH, with a reduction from 6.85 to below 4.10 witnessed in the 0–5 cm soil layer at a nitrogen application rate of 168 kg/ha. Furthermore, a substantial reduction in soil pH was manifest in the 5–10 cm layer and in the 10–15 cm layer at the maximum nitrogen application rate of 336 kg/ha [10]. The application of nitrogen fertilizers in China has resulted in the generation of an average of 20,000–221,000 moles of hydrogen ions per hectare of farmland. This represents a significant increment when compared with the 50–100 times higher production of hydrogen ions observed in acid deposition. Additionally, the neutralization capacity of salt-based ions per hectare of farmland amounts to only 15,000–20,000 moles, which is conspicuously lower than that of the hydrogen ions produced by nitrogen fertilizer application [11].

The black soil of Northeast China encompasses an area of 60,500 square kilometers, with Jilin Province accounting for 10,500 square kilometers. This soil is prevalently distributed in the central region of Jilin Province, centered on Changchun, extending southward to Siping and northward to Fuyu. The average maize yield in the region surpasses 9,000 kg per hectare. A decline in the average annual pH of black soil maize fields in this region has been noted, with a 12.12% decrease recorded in 2018 compared to 2007. In contrast, the acidity of black soil in the adjacent windbreak forest manifested a relatively minor 3.77% decline during the same period [12]. The pH of black soil in the maize belt of Jilin Province manifested a decrease of 0.87 units during the 25-year period from 1982 to 2007 [13]. Lin Han et al. noted that long-term fertilization practices led to a decrease in the pH of black soil in Jilin’s cornfields, with varying degrees of reduction [14]. Some studies have indicated that a reduction in soil pH from 7 to 5 and 4 respectively, has led to a 30.7% and 52.3% decrease in corn yields, accompanied by a 28.1% and 42.7% reduction in the number of grains per ear [15]. Furthermore, soil acidification can also result in increased prevalence of diseases and heavy metal contamination [16]. It can be rationally inferred that the acidification of black soil might exert an adverse impact on China’s food security in the future.

Soil aggregates are secondary structures formed by minerals through the combined effects of organic and inorganic substances. Their formation is influenced by multiple factors, including environmental conditions, soil management practices, and the characteristics of plants and soil [17]. Several modeling hypotheses have been put forward to account for the formation of aggregates. However, it is widely acknowledged that aggregates form through a combination of biotic and abiotic processes [18–20]. Bissonnais et al. [21] concluded that soil organic carbon, cation exchange capacity, water-soluble compounds, and organic matter secreted by roots significantly influence cluster stability. They also discovered that deeper soil clusters are less stable than surface clusters due to lower organic matter content and root-secreted substances in deeper soils. Jakšík et al. [22] investigated the influence of soil erosion on aggregate stability across different terrains and found that water stability of aggregates was negatively correlated with oxidized organic carbon and metal ion content. Their modeling study of the Water Stability aggregate Stability Index (WSA) using oxidized organic carbon content, total topographic curvature, and actual field water content as parameters showed that oxidized organic matter positively influenced the WSA index, while topographic curvature and field water content had a negative effect. In arid or semi-arid regions, calcium and magnesium carbonates re-precipitate to form secondary carbonate complexes that bind primary soil particles together [23]. Calcium ions (Ca^2+^) prevent soil particle dispersion and enhance aggregate stability by substituting sodium ions (Na^+^) and magnesium ions (Mg^2+^) in clay particles or aggregates, thereby reconsolidating disrupted aggregates [24]. Organic-inorganic complexes are soil colloids formed through complex interactions between various types of organic cementing substances and inorganic metallic cementing substances [25]. These colloids further form larger soil aggregates, so the formation of soil organic-inorganic complexes constitutes an important mechanism and material basis for stabilizing aggregates and soil fertility [26].

It has been evidenced that soil acidification leads to a reduction of cementitious substances and the leaching of saline ions, thereby diminishing organic matter activity and exerting an influence on soil aggregate formation and stability [27–29]. However, the effect of varying cementitious substances on aggregate stability is not thoroughly comprehended. A 25-year simulation of nitrogen fertilizer application was conducted on soil columns. The incubation test analyzed the variations in main cementing substances under acidification conditions. Additionally, the changes in soil microaggregate stability at different acidification levels were evaluated using ultrasonic crushing and heat difference analysis. The study aimed to elucidate the relationship between the stability energy of microaggregates and the main cementing substances of the soil. These findings will provide a theoretical basis for alleviating and preventing black soil acidification in Jilin Province.

## 2 Materials and methods

### 2.1 Test soil

**Fig 1:**
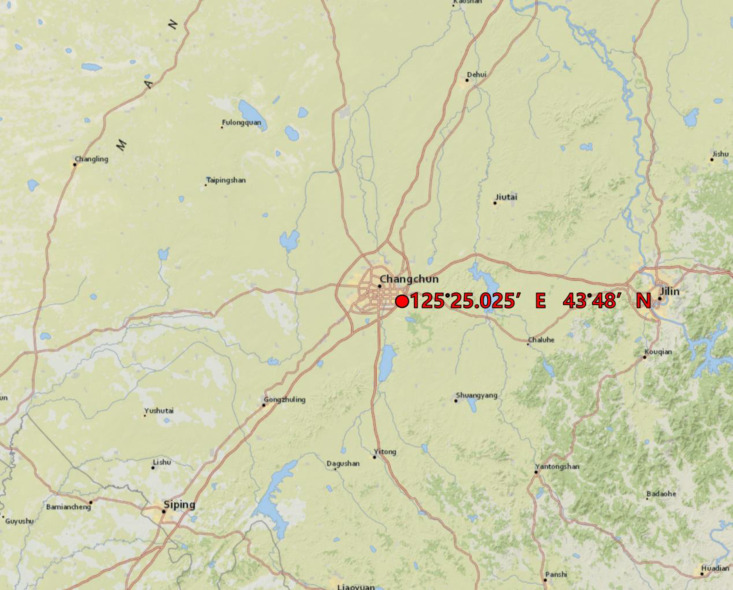
The red circle in the map shows the soil collection site, which is located in the southeast of Jilin Agricultural University, Changchun, Jilin Province, China. Map resources from USGS EROS (Earth Resources Observatory and Science (EROS) Center) (public domain): https://www.usgs.gov/products/maps/map-releases.

The soil column simulation test constitutes a widely utilized methodology for the investigation of soil acidification processes [30,31]. A soil column acidification culture test was conducted on soil collected in September 2018 from the southeast of Jilin Agricultural University (125°25.025′E, 43°48′N), shown in Fig 1. The soil, classified as black soil (The United Nations World Soil Atlas is categorized as phaiozem). The depth for soil collection ranges from 0 to 20 cm. Before collection, the land surface was cleared of stones, dead leaves, and other impurities. Care was taken to avoid compressing the soil during collection to maintain aggregate stability. Compressed soil edges were removed as much as possible. The soil had the following properties: The soil is characterised by a dark black color, softness, elasticity, fragility, and a high concentration of clay particles, organic matter content of 29.12 g/kg, pH of 6.48, and cation exchange capacity of 29.5 cmol/kg, the base saturation is 99.26%.

The soil column preparation was conducted as follows: After the soil was mixed, it was sieved into particle sizes of 10–7 mm, 7–5 mm, 5–2 mm, 2–1 mm, and >1 mm. The proportions were 8.83%, 13.82%, 25.9%, 21.52%, and 29.97%, respectively. The soil was then uniformly mixed and divided into 30 portions, each weighing 1045 g. Soil columns were prepared to ensure that each contained the same proportion of black soil particles. The columns were 40 cm in length and 9 cm in width, with an inner diameter of approximately 8 cm. The tube were constructed from PVC and was sealed at the bottom with two layers of 30-mesh nylon mesh. Thirty grams of quartz sand was evenly spread at the bottom of the PVC tube, and an 80-mesh nylon mesh slightly larger than the inner diameter of the tube was placed on top. Each PVC tube was filled with 1045 g of soil to ensure a consistent particle distribution in each column. The soil was gently filled into tube, and a 30-mesh nylon mesh was placed on top. A plastic bag or bucket was placed at the bottom to collect the drenching liquid.

The soil column acidification culture test was divided into six treatment groups, namely: the control group (Group CK), which received no fertilizer to eliminate the potential impact of leaching; the simulated 5-year fertilization group (Group A); the simulated 10-year fertilization group (Group B); the simulated 15-year fertilization group (Group C); the simulated 20-year fertilization group (Group D); and the simulated 25-year fertilization group (Group E). Each group consisted of five soil columns, and each soil column was a replicate, that is, each group had five replicates. Following typical fertilization practices in the black soil area of Jilin, urea was used as the nitrogen fertilizer and diammonium phosphate as the phosphorus fertilizer (due to varying phosphorus content in superphosphate, making quantitative research challenging). Investigations have demonstrated that the amounts of nitrogen and phosphorus applied to cornfields in the black soil region of central Jilin Province are 217.3 and 84.2 kg/hm², respectively. The quantity of nitrogen and phosphorus applied to cornfields in the vicinity of the sampling site is 220 and 80 kg, respectively. Hence, the fertilizer dosage was set at 220 kg of nitrogen per hectare and 80 kg (P_2_O_5_) of phosphorus. Due to significant ammonia volatilization and leaching in the soil column compared to the field, the dosage was increased by 1.5 times, resulting in 1.27 g of urea and 0.48 g of diammonium hydrogen phosphate per soil column. The fertilizers were dissolved in 360ml distilled water and uniformly added to the soil columns using the dropping method. The process lasted for approximately about 2 hours.

Ammonia volatilization will become evident from the fourth day and will typically persist until the fifteenth day or so [32]. In order to ensure that the nitrogen turnover after entering the soil reaches a stable state, the acidification time is set at 20 days per cycle,that is, a solution equivalent to five years of fertilization is initially added to the soil, followed by an incubation period of 20 days. The annual rainfall in the central region of Jilin Province is 586.85 mm. However, in order to prevent excessive water accumulation on the soil surface during leaching, the simulated rainfall is reduced to 400 ml.

**Fig 2:**
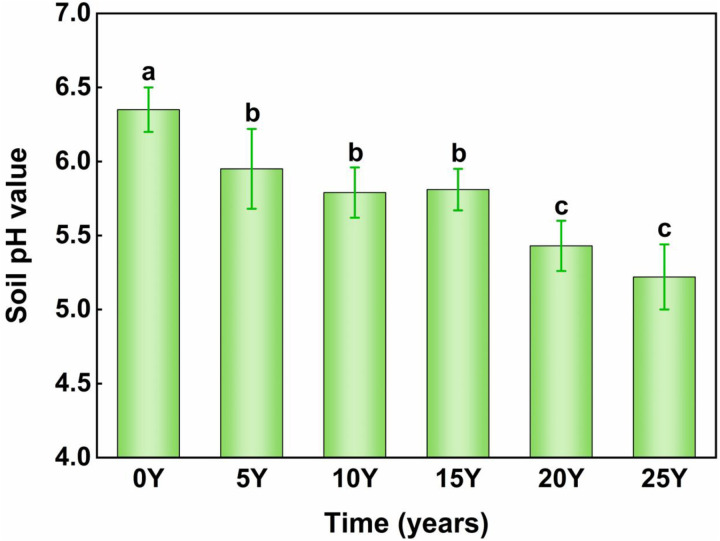
Regarding the acidity of black soil tested across various fertilization durations, the control group (0Y) received no fertilization, while the treatment groups included soils that had been fertilized for 5 years (5Y), 10 years (10Y), 15 years (15Y), 20 years (20Y), and 25 years (25Y).

The simulation test encompass five rounds. In the first round, the CK group was excluded while the other groups received a solution equivalent to 5 years of fertilizer. The control group was given an equal amount of distilled water. After 20 days of cultivation, a 5-day drenching treatment was carried out, with 400 ml of distilled water applied daily via drip irrigation. After drenching, the soil columns were baked at 34°C for 48 hours to rapidly reduce water content. The soil was then gently knocked, carefully poured out, mixed, and reloaded into PVC pipes for the next round of acidification simulation testing. In the second round, groups B, C, D, and E received another 5-year fertilizer amount. Groups A and B were drenched with distilled water. After a 20-day incubation, the same 5-day drenching test was conducted. And so on until the fertilizer solution had been added to the Group E soil for a total of 25 years. The changes in black soil acidity with varying degrees of acidification are shown in Fig 2.

### 2.2 Sample analysis

The pH was determined by potentiometric method; soil organic matter and water-soluble organic carbon were determined by the potassium dichromate volumetric method and the polyhydric soil-ratio method, respectively [33]; Exchangeable calcium and magnesium were determined by the ammonium acetate leaching-atomic absorption method [34]; and CaCO_3_ was determined by the gasometric method [35].

In order to reduce the bias, when measuring inorganic composite cementing substances, organic-inorganic composite cementing substances and soil aggregate energy, the tested soil of 5 soil columns in each group was evenly mixed and repeated for three times. When correlation analysis is performed, the same treatment is used to determine the organic composite cementing substances.

The determination of aggregate stability energy E is based on the energy required to disrupt 99% of soil aggregates (considered fully broken), energy can be evaluated by ultrasonic crushing-thermal difference approach [36]. This approach employs a 10% ethanol solution rather than distilled water to mitigate interference. The ultrasonic crusher model is JY-96IIN, with a probe amplitude rod diameter of 6 mm. The ultrasonic vibrator amplitude gear is set to 6 mm, the ultrasonic power to 50%, the probe is 20 mm deep below the liquid surface, and the ultrasonic output power is about 4.8 W.

Following ultrasonic treatment, clay particles (less than 2 um), silt particles (2–53 um), and sand particles (53–200 um) were obtained respectively via wet sieve and straw methods.

The dispersion and fragmentation dynamic characteristics of water-stable aggregates were fitted and characterized by SDCC, ADCC, and ALDC equations. The critical energy Ecrit for pulverized particles was computed based on the fragmentation characteristic curve of aggregates [37]:


$$SDCC=C_{C}+C_{0}[1-\exp(-k_{c}t)]$$ (1)

SDCC denotes the dynamic characteristic curve of soil clay, where C_0_ represents the clay mass fraction prior to the soil being treated by ultrasonic waves, C_C_ is the variable of the clay mass fraction during the ultrasonic dispersion process, C_0_ + C_C_ is the clay mass fraction after the soil is completely broken by ultrasonic wave treatment, and k_c_ is the rate constant of aggregate breaking.


$$ADCC=A_{C}+A_{0}\exp(-k_{a}t)$$ (2)

ADCC denotes the dynamic characteristic curve of soil sand particles. A_0_ indicates the mass fraction of sand particles before the aggregate is fractured by ultrasonic waves, A_C_ - A_0_ is the variable of the mass fraction of sand particles during the ultrasonic wave crushing process, A_C_ is the mass fraction of clay particles after the soil is completely broken by ultrasonic wave treatment, and k_a_ is the crushing rate constant of aggregates.


$$ALDC=C_{0}\exp(-k_{c}t)-A_{0}\exp(-k_{a}t)+B_{c}$$ (3)

ALDC denotes the kinetic characteristic curve of powder particles and B_C_ is the mass fraction of powder particles. The curve is divided into two parts: the release of powder particles by sand particles and the fragmentation of powder particles into clay particles. Critical stabilization energy Ecrit is the energy required when the increase speed and crushing speed of powder particles reach a balance, which is computed by the following formula:


$$E_{crit}=\frac{\ln(k_{c}/k_{a})}{k_{c}-k_{a}}$$ (4)

### 2.3 Data processing

The data were calculated and analysed using WPS Office, the kinetic equation for the dispersion and fragmentation of aggregates was fitted using Origin 2021, the correlation between the main cementing substances in the black soil and the stability of aggregates was analysed, and SPSS Statistics 21.0 was used to process the analysis of the weights of the independent variables in the multiple linear regression and to diagnose collinearity.

## 3 Results and discussion

### 3.1 Effects of black soil acidification processes on organic carbon

**Fig 3:**
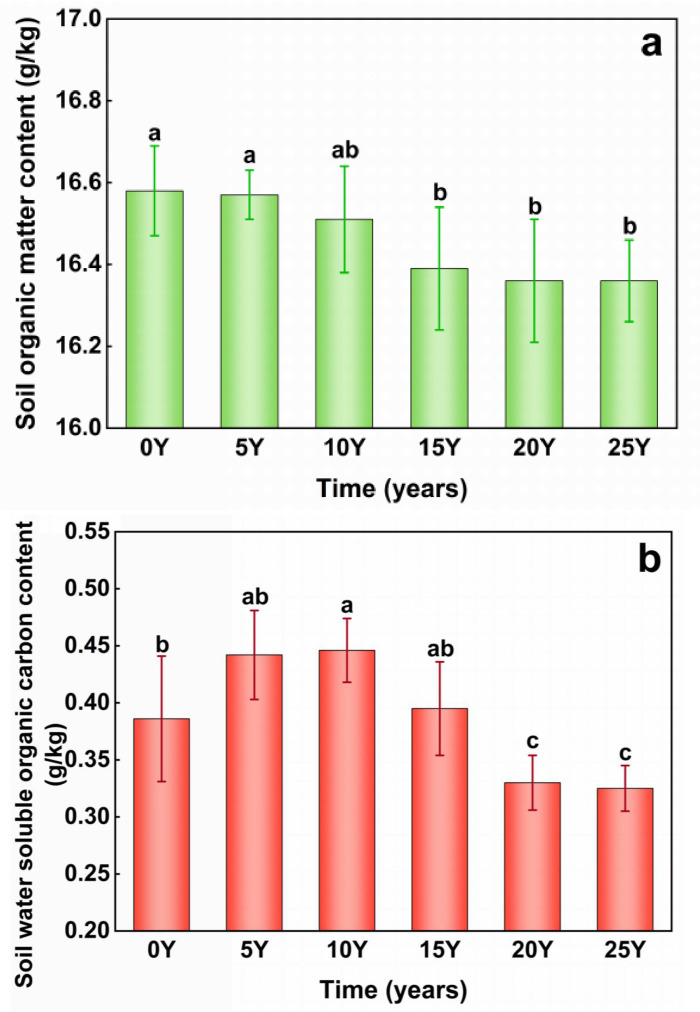
(a) Changes of SOC (Soil organic matter) content during acification of black soil. The control group (0Y) received no fertilization, while the treatment groups included soils that had been fertilized for 5 years (5Y), 10 years (10Y), 15 years (15Y), 20 years (20Y), and 25 years (25Y). (b) Changes of WSOC (Soil water soluble organic matter) content during acification of black soil. The control group (0Y) received no fertilization, while the treatment groups included soils that had been fertilized for 5 years (5Y), 10 years (10Y), 15 years (15Y), 20 years (20Y), and 25 years (25Y).

Soil organic carbon (SOC) constitutes an important component that significantly contributes to soil cementation. It exerts positive effects on water-stabilized aggregates and enhances the soil’s acid buffering capacity [38]. As shown in Fig 3(a), soil organic carbon (SOC) gradually declined with the reduction in soil pH. The pH decreased from 6.35 to 5.81, and SOC content dropped from 16.58 g/kg to 16.39 g/kg. The most significant decline in SOC occurred when the pH dropped from 5.79 to 5.81, followed by a 0.06 g/kg decrease when the pH fell from 5.95 to 5.79. No significant change in SOC was observed when the pH decreased from 5.81 to 5.22. After 25 years of fertilization and acidification incubation, SOC decreased by a total of 0.23 g/kg, averaging an annual reduction of 0.01 g/kg. For every 0.1 unit decrease in pH, SOC decreased by 0.02 g/kg. Correlation analysis showed a strong consistency between the decline in SOC and the decrease in pH. A significant decrease in pH was accompanied by a significant decrease in SOC, as also demonstrated by Chen Lin et al. in their 22-year study of meadow black soil, which found that the pH and organic matter content were lowest in the single fertilizer group compared to other treatments [14].

As shown in Fig 3(b), the Water-soluble organic carbon (WSOC) of the test soil displayed a distinct pattern as pH decreased: an initial increase, followed by a decline and subsequent stabilization. The soil pH decreased from 6.35 to 5.95 during the 10-year fertilizer acidification incubation stage, which was marked by a WSOC increase of 0.056 g/kg. When the pH decreased from 5.79 to 5.43, WSOC significantly decreased by approximately 0.115 g/kg. From the 20th to the 25th year of simulated fertilizer acidification cultivation, no significant changes in pH or WSOC content were observed. Overall, WSOC decreased by 0.06 g/kg during the entire fertilizer acidification incubation experiment. The findings indicated that soil acidification can reduce WSOC, showing an initial increase followed by a decline. This pattern may be due to the initial fertilizer application increasing soil nitrogen and phosphorus, enhancing microbial activity, and accelerating SOC mineralization, leading to increased WSOC during the early and intermediate stages. However, as soil pH declined, microbial activity was suppressed, SOC mineralization slowed, and WSOC production gradually decreased [38,39].

### 3.2 Influence of black soil acidification process on organic-inorganic composite cementing substances

**Fig 4:**
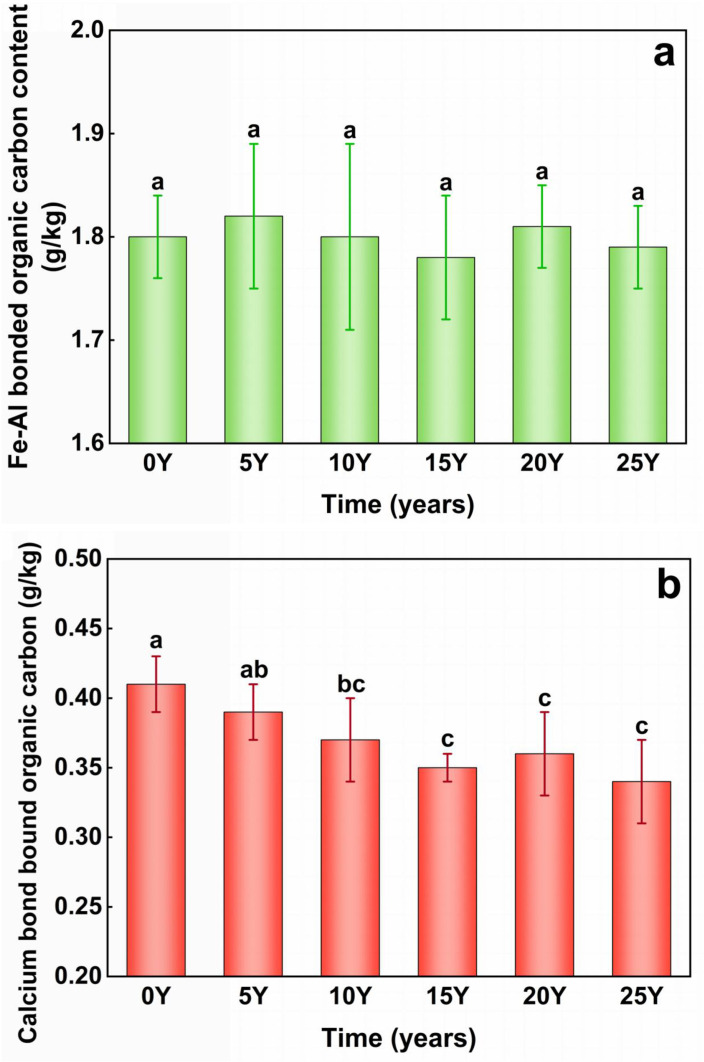
(a) Changes of Fe-Al bond-bound soil organic carbon (Fe/Al-SOC) content during acification of black soil. (b) Changes of Calcium bond-bound soil organic carbon (Ca-SOC) content during acification of black soil. The control group (0Y) received no fertilization, while the treatment groups included soils that had been fertilized for 5 years (5Y), 10 years (10Y), 15 years (15Y), 20 years (20Y), and 25 years (25Y).

The term “organic-inorganic complex bound soil organic carbon” encompasses two main types: calcium bond-bound soil organic carbon (Ca-SOC) and iron and aluminum bond-bound soil organic carbon (Fe/Al-SOC). These are regarded as inert organic carbon with slow turnover and strong stability, playing a crucial role in regulating soil carbon pools [40]. Free aluminum oxides are persistent cementing substances [41]. The continuous decrease in pH did not exert a significant impact on the Fe/Al-SOC content of the soil (Fig 4(a)), which remained stable around 1.80 g/kg during the simulated fertilizer-acidification incubation. Fig 4(b) shows the calcium bond-bound soil organic carbon content of the tested soils under varying acidity levels. This content showed a declining trend with decreasing pH, from 0.41 g/kg to 0.34 g/kg, with an average annual reduction of 0.029 g/kg. Notably, the calcium bond-bound soil organic carbon content of the black soil significantly declined during the pH stage from 6.35 to 5.79.

### 3.3 Influence of the acidification process of black soil on inorganic cementing substances

**Fig 5:**
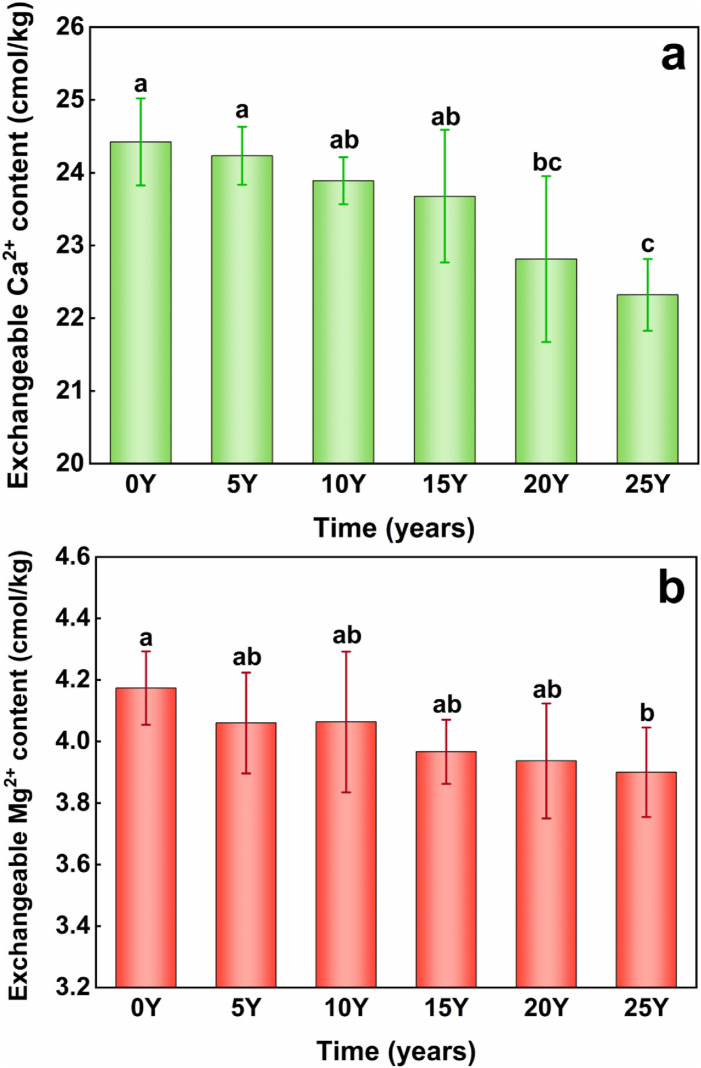
(a) Changes of exchangeable Ca^2+^ content during acification of black soil. (b) Changes of exchangeable Mg^2+^ content during acification of black soil. The control group (0Y) received no fertilization, while the treatment groups included soils that had been fertilized for 5 years (5Y), 10 years (10Y), 15 years (15Y), 20 years (20Y), and 25 years (25Y).

The inorganic cementing substances of soil mainly comprise iron and aluminum oxides, calcium carbonate, and clay minerals such as exchange calcium and magnesium ions.These substances exchange calcium and magnesium ions, which act as cementing agents to bond organic matter with mineral clay particles, creating a composite of organic and inorganic materials. This process is of vital importance for the formation and stabilization of soil aggregates [42]. Consequently, exchanged calcium and magnesium ions are considered soil inorganic cementing substances. As shown in Fig 5(a) and 5(b), there is a negative correlation between exchangeable calcium and magnesium levels and soil acidification. The pH dropped significantly from 6.35 to 5.22, along with a substantial decrease in exchangeable magnesium levels to 0.27 cmol/kg. The differences in other treatments did not reach statistical significance. A significant positive correlation between black soil pH and exchangeable Ca^2+^ and Mg²⁺ was identified subsequent to long-term nitrogen fertilizer application^14^. The pH decreased from 6.35 to 5.81, exchangeable Ca^2+^ changes were not statistically significant. However, when pH levels dropped below 5.43, changes in exchangeable Ca^2+^ were significant. declined by approximately 1.61 cmol⁻¹. At the lowest pH of 5.22, the decline reached 2.01 cmol⁻¹. For each 0.1 unit decrease in pH, the decline in exchangeable Ca^2+^ was 2.01 cmol⁻¹. A one-unit pH decrease caused a 0.19 g/kg reduction in exchangeable Ca^2+^ and a 0.02 g/kg reduction in exchangeable Mg²⁺.

**Fig 6:**
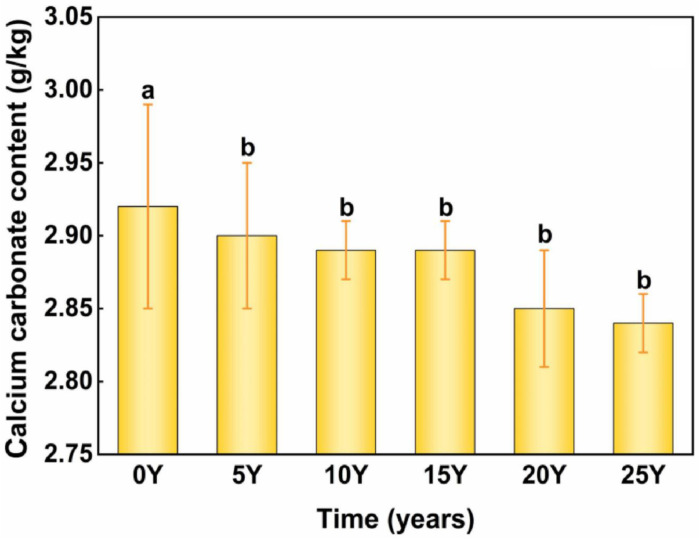
Changes of exchangeable calcium carbonate content during acification of black soil. The control group (0Y) received no fertilization, while the treatment groups included soils that had been fertilized for 5 years (5Y), 10 years (10Y), 15 years (15Y), 20 years (20Y), and 25 years (25Y).

The low CaCO₃ content in the test soil, with an initial concentration of 2.92 g/kg, was attributed to the high annual precipitation at the sampling site, which made it difficult for CaCO₃to accumulate in the top 20 cm of the soil [43]. As is shown in Fig 6, during the simulated fertilization acidification incubation stage, a significant negative correlation between soil CaCO₃ content and pH was observed, leading to a decrease in CaCO₃ content from 2.92 g/kg to 2.84 g/kg. Additionally, for every 0.1 unit decrease in pH, CaCO₃ content decreased by an average of 0.007 g/kg.

### 3.4 Influence of black soil acidification process on the stabilizing energy of aggregates

**Fig 7:**
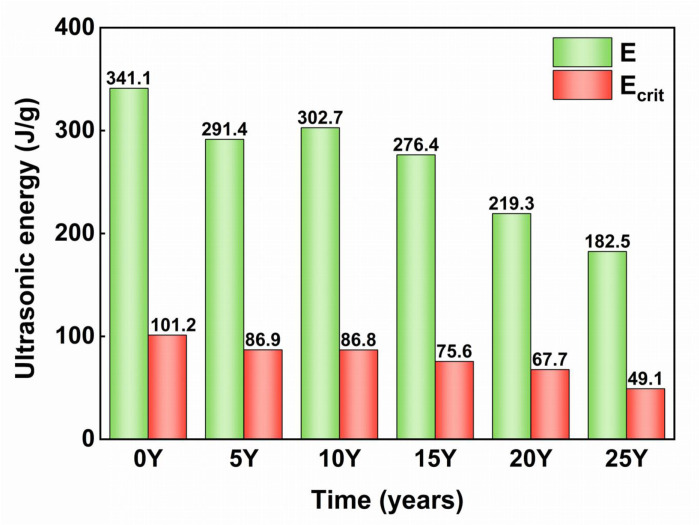
E (soil aggregate stability energy) is the amount of energy required when 99% of the soil is disrupted, Ecrit (soil critical stabilization energy) is the energy required when the increase speed and crushing speed of powder particles reach a balance. The control group (0Y) received no fertilization, while the treatment groups included soils that had been fertilized for 5 years (5Y), 10 years (10Y), 15 years (15Y), 20 years (20Y), and 25 years (25Y).

When 99% of the aggregate is disrupted by ultrasound, the aggregate is regarded as being in a completely broken state. As shown in Fig 7, the energy required to fragment 99% of the water-stable microaggregates of soils at different acidification stages was 341.1 J/g, 291.4 J/g, 302.7 J/g, 276.4 J/g, 219.3 J/g, and 182.5 J/g. Acidification decreased the E of these microaggregates by 158.6 J/g, epresenting a 46.5% reduction. On average, a 0.1 unit decrease in pH led to a reduction of 14.04 J/g in E.

Ecrit decreases as the acidification of the test black soil increases. Ecrit decreased from 101.2 J/g to 49.1 J/g, epresenting a 51% reduction. On average, Ecrit decreased by 48% for every 0.1 unit drop in pH. This suggests that sand grains lose stability more rapidly than powder grains with increasing acidification. This difference in stability is probably attributed to the higher organic matter content within powder grains as opposed to sand grains. Zhang et al [44]. found that black soil’s powder grains contain significantly more organic matter than sand grains. Soil organic carbon (SOC) is of crucial importance for acid buffering in soil due to its high number of carboxyl groups. These groups increase the soil’s negative charge, enhancing the adsorption of salinity and aluminum ions and improving acid buffering [39,45]. In Al-buffered soils with an initial pH below 4.2, SOC continues to adsorb exchangeable cations, thereby inhibiting acidification [38]. Therefore, SOC improves the anti-acidification stability of water-stable aggregates and mitigates the decline in E.

### 3.5 Relationship between aggregate stability and cementing substances during acidification of black soil

The acidification of black soil altered the primary cementing substances and the stability of soil aggregates. Most of these alterations significantly correlated with soil acidity. Cementing substances are essential for forming and stabilizing aggregates. Thus, the relationship between cementing substances and aggregate stability under prolonged acidification highlights the main factors driving aggregate disintegration during this process.

**Fig 8:**
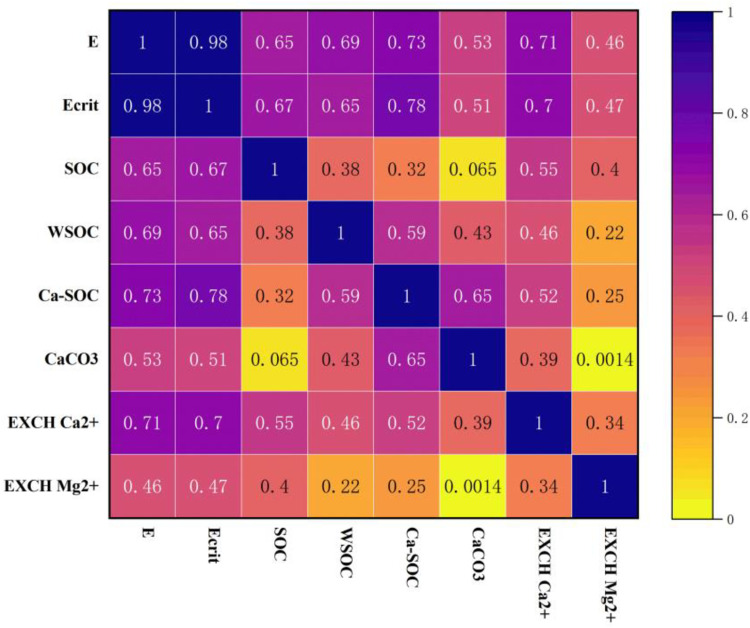
The higher the value, the stronger the correlation.

Fig 8 shows the correlation analysis between primary cementing substances and aggregate energy in the test soil. The energy E of the test soil exhibited a highly significant positive correlation with SOC, WSOC, Ca-SOC, and exchanged Ca^2+^, a significant positive correlation with CaCO_3_, and no correlation with exchanged Mg^2+^. The the most significant correlation was with Ca-SOC, followed by exchanged Ca^2+^, WSOC, and SOC. Ecrit also demonstrated highly significant positive correlations with SOC, WSOC, Ca-SOC, and exchangeable Ca^2+^, and significant positive correlations with CaCO_3_ and exchangeable Mg^2+^. The strongest correlation was with Ca-SOC, followed by exchangeable Ca^2+^.

Table 1 presents the results of a multiple linear regression analysis on the weight operation and covariance diagnosis. SOC significantly impacts the reduction of E in the test soil, with a standard coefficient of 0.312. Ca-SOC has the second greatest impact on Ecrit, with a standard coefficient of 0.331. This study shows that organic matter is crucial in forming and stabilizing aggregates [46,47]. Initially, organic matter serves as the main agent in the formation of aggregates or unstable large aggregates. After crushing, smaller aggregates form within the larger ones and are released. These smaller aggregates then combine with cementing materials to form larger aggregates [48,49].

**Table 1:** Multiple regression analysis of aggregate stability and cementing substance in black soil during acidification.

Treatment	cementing material	Standardized Coefficients	correlation coefficient
E	SOC	0.312	0.002
	Ca-SOC	0.236	0.000
	WSOC	0.229	0.001
	CaCO_3_	0.187	0.012
	Ca^2+^	0.181	0.000
	Mg^2+^	0.165	0.027
Ecrit	Ca-SOC	0.401	0.000
	SOC	0.331	0.001
	Mg^2+^	0.154	0.025
	Ca^2+^	0.153	0.000
	WSOC	0.129	0.002
	CaCO_3_	0.115	0.015

Increasing organic carbon content improves aggregate stability [50], and organic matter content shows a highly significant positive correlation with aggregate stabilization energy. Zhu et al. [51] conducted an incubation study adding various exogenous organic matters to clayey soils. The ultrasonic crushing test results indicated a significant enhancement in soil aggregate stabilization energy after adding organic matter. However, some studies suggest that lower pH levels can impede soil microbial activity, limiting organic matter decomposition and causing soil carbon accumulation [52,53]. This effect of pH on organic matter depends on factors such as soil type, fertilizer use, and climate [54].The primary factor causing the decrease in Ecrit in the test soil was the reduction of Ca-SOC content, with a standardized coefficient of 0.401. The contribution to E was ranked second, following SOC, with a standardized coefficient of 0.236. This study showed that calcium positively affects soil aggregate stabilization. The loss of Ca^2+^ leads to an increase in soil aggregate porosity and decreased stability [50]. Ca^2+^ aids in aggregate formation and stability through coordination reactions, cation bridging, and forming cementing substances with carboxyl, carbonyl, and phenolic hydroxyl groups in the soil. Additionally, calcium increases the hydrophobicity of aggregates [55]. The impact of calcium on the fixation of soil organic carbon and aggregates is more significant than that of salt-based cations such as sodium, potassium, and magnesium [56–58].

The multiple regression analysis indicated that the reduction of exchangeable Ca^2+^ also contributed to decreased aggregate stability. However, it has been demonstrated that the exchangeable Ca^2+^ content has increased in black soils, despite significant acidification following years of agricultural production [59]. This may be due to the extensive use of calcium-containing fertilizers. Exchangeable Mg^2+^ facilitates the formation of water-stable aggregates by reducing the electrostatic force between humus and clay particles [60].Therefore, the decline in exchangeable Mg^2+^ is a primary factor in reducing E.

SOC and Ca-SOC are highly sensitive to changes in soil acidity and aggregate stabilization energy. Thus, they can be used as indicators of soil acidification and the decline in E, especially in the early stages of acidification.

## 4. Conclusion

The main cementing substances in the test soils—SOC, WSOC, Ca-SOC, exchangeable Ca^2+^, exchangeable Mg^2+^, and CaCO₃—showed a significant positive correlation with pH. After 25 years of soil column acidification incubation, their contents decreased by 0.22 g/kg, 0.06 g/kg, 1.12 g/kg, 2.10 g/kg, 0.27 g/kg, and 2.84 g/kg, respectively.

The ultrasonic crushing heat difference test showed that the microaggregate stabilization energy of the black soil decreased with increasing soil acidity. This led to a reduction in aggregate stability energy (E) from 341.1 J/g to 182.5 J/g, a total decrease of 158.6 J/g. Additionally, the critical stabilization energy (Ecrit) of the soil particlesdeclined by 51.48%.

The reduction in SOC content had the greatest impact on the decline in soil aggregate stability energy (E), followed by Ca-SOC. Conversely, Ca-SOC had the most influence on the reduction in sand grain critical stabilization energy (Ecrit), followed by SOC.

## Supporting information

S1 Data Supplemental raw data. (DOCX)
