# Supplementary material for: The impact of soil acidification on cementing substances and aggregate stability
Source: PLoS One. 2025 Apr 16;20(4):e0318417. doi: 10.1371/journal.pone.0318417 (PMC12002804; doi:10.1371/journal.pone.0318417)
Supplement: S1 Data — (DOCX) [file pone.0318417.s001.docx]

Experimental data unit of this study

**In the “2 Materials and methods” section**

**Fig. 2** Soil pH value under different simulated years of fertilization.

| Changes of soil pH in different simulated fertilization-leaching experiment periods | | | | | | |
| --- | --- | --- | --- | --- | --- | --- |
| treatment | CK(0Y) | 5Y | 10Y | 15Y | 20Y | 25Y |
| Mean value | 6.35 | 5.95 | 5.79 | 5.81 | 5.43 | 5.22 |
| deviation | 0.15 | 0.27 | 0.17 | 0.14 | 0.17 | 0.22 |

CK(0Y):No fertilizer application; 5Y:Simulated fertilizer application for 5 years; 10Y:Simulated fertilizer application for 10 years; 15Y: Simulated fertilizer application for 15 years; 20Y:Simulated fertilizer application for 20 years; 25Y:Simulated fertilizer application for 25 years

**In the “3.1 Effects of black soil acidification processes on organic carbon” section**

**Fig. 3(a)** Soil organic matter content of black soil under different acidity.

| Changes of SOC in different simulated fertilization-leaching experiment periods(g/kg) | | | | | | |
| --- | --- | --- | --- | --- | --- | --- |
| treatment | CK(0Y) | 5Y | 10Y | 15Y | 20Y | 25Y |
| Mean value | 16.58 | 16.57 | 16.51 | 16.39 | 16.36 | 16.36 |
| deviation | 0.11 | 0.06 | 0.13 | 0.15 | 0.15 | 0.10 |

**Fig. 3(b)** Water-soluble organic carbon content of black soil under different acidity.

| Changes of WSOC pH in different simulated fertilization-leaching experiment periods(g/kg) | | | | | | |
| --- | --- | --- | --- | --- | --- | --- |
| treatment | CK(0Y) | 5Y | 10Y | 15Y | 20Y | 25Y |
| Mean value | 0.39 | 0.44 | 0.45 | 0.39 | 0.33 | 0.33 |
| deviation | 0.06 | 0.04 | 0.03 | 0.04 | 0.02 | 0.02 |

**In the “3.2 Influence of black soil acidification process on organic-inorganic composite cementing substances” section**

**Fig. 4(a)** Iron and aluminum bond-bound soil organic carbon (Fe/Al-SOC) content of black soil under different acidity.

| Changes of Fe/Al-SOC in different simulated fertilization-leaching experiment periods(g/kg) | | | | | | |
| --- | --- | --- | --- | --- | --- | --- |
| treatment | CK(0Y) | 5Y | 10Y | 15Y | 20Y | 25Y |
| Mean value | 1.80 | 1.82 | 1.80 | 1.78 | 1.81 | 1.79 |
| deviation | 0.04 | 0.07 | 0.09 | 0.06 | 0.04 | 0.04 |

**Fig. 4(b)** Calcium bond-bound soil organic carbon (Ca-SOC) content of black soil under different acidity.

| Changes of Ca-SOC in different simulated fertilization-leaching experiment periods(g/kg) | | | | | | |
| --- | --- | --- | --- | --- | --- | --- |
| treatment | CK(0Y) | 5Y | 10Y | 15Y | 20Y | 25Y |
| Mean value | 0.41 | 0.39 | 0.37 | 0.35 | 0.36 | 0.34 |
| deviation | 0.02 | 0.02 | 0.03 | 0.01 | 0.03 | 0.03 |

**In the “3.3 Influence of the acidification process of black soil on inorganic cementing substances” section**

**Fig. 5(a)** Exchanged calcium ions content of black soil under different acidity.

| Changes of exchangeable Ca²⁺ in different simulated fertilization-leaching experiment periods (cmol/kg) | | | | | | |
| --- | --- | --- | --- | --- | --- | --- |
| treatment | CK(0Y) | 5Y | 10Y | 15Y | 20Y | 25Y |
| Mean value | 24.42 | 24.23 | 23.89 | 23.68 | 22.81 | 22.32 |
| deviation | 0.60 | 0.40 | 0.32 | 0.91 | 1.14 | 0.49 |

**Fig. 5(b)** Exchanged magnesium ions content of black soil under different acidity.

| Changes of exchangeable Mg²⁺ in different simulated fertilization-leaching experiment periods (cmol/kg) | | | | | | |
| --- | --- | --- | --- | --- | --- | --- |
| treatment | CK(0Y) | 5Y | 10Y | 15Y | 20Y | 25Y |
| Mean value | 4.17 | 4.06 | 4.06 | 3.97 | 3.94 | 3.90 |
| deviation | 0.12 | 0.16 | 0.23 | 0.10 | 0.19 | 0.15 |

**Fig. 5(c)** The CaCO₃ content content of black soil under different acidity.

| Changes of CaCO3 in different simulated fertilization-leaching experiment periods (cmol/kg) | | | | | | |
| --- | --- | --- | --- | --- | --- | --- |
| treatment | CK(0Y) | 5Y | 10Y | 15Y | 20Y | 25Y |
| Mean value | 2.92 | 2.90 | 2.89 | 2.89 | 2.85 | 2.84 |
| deviation | 0.07 | 0.05 | 0.02 | 0.02 | 0.04 | 0.02 |

**In the “3.4 Influence of black soil acidification process on the stabilizing energy of aggregates” section**

Dynamic characteristic curve equation of soil under different acidity.

|  | SDCC dynamic characteristic curve equation of soil under different acidity ($SDCC=C_{C}+C_{0}[1-\exp(-k_{c}t)]$) |
| --- | --- |
| CK(0Y) | 0.14608+0.48692[1-exp(-0.00661*t)] |
| A(5Y) | 0.01678+0.47582[1-exp(-0.00748*t)] |
| B(10Y) | 0.01615+0.46385[1-exp(-0.00788*t)] |
| C(15Y) | 0.01769+0.47627[1-exp(-0.00959*t)] |
| D(20Y) | 0.02229+0.45919[1-exp(-0.00918*t)] |
| E(25Y) | 0.02687+0.43922[1-exp(-0.01504*t)] |

|  | ADCCdynamic characteristic curve equation of soil under different acidity($ADCC=A_{C}+A_{0}\exp(-k_{A}t)$) |
| --- | --- |
| CK(0Y) | 0.1142+0.63627exp(-0.01408*t) |
| A(5Y) | 0.13138+0.52999exp(-0.01678*t) |
| B(10Y) | 0.13719+0.55451exp(-0.01615*t) |
| C(15Y) | 0.13502+0.54613exp(-0.01769*t) |
| D(20Y) | 0.12936+0.526471exp(-0.02229*t) |
| E(25Y) | 0.13105+0.50729exp(-0.02687*t) |

|  | ALDCdynamic characteristic curve equation of soil under different acidity($ALDC=C_{0}\exp(-k_{c}t)-A_{0}\exp(-k_{A}t)+B_{c}$) |
| --- | --- |
| CK(0Y) | 0.48692*exp(-x*0.00661) - 0.63627*exp(-x*0.01408) + A |
| A(5Y) | 0.47582*exp(-x*0.00748) - 0.55451*exp(-x*0.01615) + A |
| B(10Y) | 0.46385*exp(-x*0.00788) - 0.52999*exp(-x*0.01678) + A |
| C(15Y) | 0.47627*exp(-x*0.00959) - 0.54613*exp(-x*0.01769) + A |
| D(20Y) | 0.45919*exp(-x*0.00918) - 0.52647*exp(-x*0.02229) + A |
| E(25Y) | 0.43922*exp(-x*0.01504) - 0.50729*exp(-x*0.02687) + A |

The parameters of the dynamic equation of soil aggregates breaking under different acidity.

| Soil | Cc/g g-1 | C0/g g-1 | Cc+Co/g g-1 | Ao/g g-1 | Ac/g g-1 | Ao-Ac/g g-1 | B0/g g-1 |
| --- | --- | --- | --- | --- | --- | --- | --- |
| CK(0Y) | 0.14608 | 0.48692 | 0.63137 | 0.63627 | 0.1142 | 0.5221 | 0.26284 |
| A(5Y) | 0.01678 | 0.47582 | 0.63972 | 0.52999 | 0.13138 | 0.3986 | 0.22295 |
| B(10Y) | 0.01615 | 0.46385 | 0.63278 | 0.55451 | 0.13719 | 0.4173 | 0.23705 |
| C(15Y) | 0.01769 | 0.47627 | 0.63258 | 0.54613 | 0.13502 | 0.4111 | 0.23133 |
| D(20Y) | 0.02229 | 0.45919 | 0.64524 | 0.526471 | 0.12936 | 0.3971 | 0.21855 |
| E(25Y) | 0.02687 | 0.43922 | 0.62022 | 0.50729 | 0.13105 | 0.3762 | 0.23979 |

C_0_ represents the clay mass fraction prior to the soil being treated by ultrasonic waves; C_C_ is the variable of the clay mass fraction during the ultrasonic dispersion process; C_0_ + C_C_ is the clay mass fraction after the soil is completely broken by ultrasonic wave treatment; A_0_ indicates the mass fraction of sand particles before the aggregate is fractured by ultrasonic waves; A_C_ - A_0_ is the variable of the mass fraction of sand particles during the ultrasonic wave crushing process; A_C_ is the mass fraction of clay particles after the soil is completely broken by ultrasonic wave treatment; B_C_ is the mass fraction of powder particles.

**Fig. 6** The determination of aggregate stability energy (E) and Critical stabilization energy (Ecrit) of soil under different acidity.

| Soil | kc/s-1 | ka/s-1 | **E/J g-1** | **Ecrit/J g-1** |
| --- | --- | --- | --- | --- |
| CK(0Y) | 0.00661 | 0.01408 | 341.1 | 101.2 |
| A(5Y) | 0.00748 | 0.01678 | 291.4 | 86.9 |
| B(10Y) | 0.00788 | 0.01615 | 302.7 | 86.8 |
| C(15Y) | 0.00959 | 0.01769 | 276.4 | 75.6 |
| D(20Y) | 0.00918 | 0.02229 | 219.3 | 67.7 |
| E(25Y) | 0.01504 | 0.02687 | 182.5 | 49.1 |

k_c_ is the rate constant of aggregate breaking;k_a_ is the crushing rate constant of aggregates.

**In the “3.5 Relationship between aggregate stability and cementing substances during acidification of black soil” section**

| **Fig. 7** Correlation analysis between primary cementing substances and aggregate energy in the test soil. | | | | | | | | |
| --- | --- | --- | --- | --- | --- | --- | --- | --- |
| treatment | E/J g- | Ecrit/J g-1 | SOC | WSOC | Ca-SOC | CaCO3 | exchangeable Ca2+ | exchangeable Mg2+ |
| CK（0Y） | 341.1 | 101.2 | 28.50 | 0.46 | 0.44 | 2.93 | 23.59 | 4.04 |
|  | 341.1 | 101.2 | 28.54 | 0.36 | 0.41 | 2.99 | 24.72 | 4.33 |
|  | 341.1 | 101.2 | 28.87 | 0.38 | 0.38 | 2.83 | 24.96 | 4.15 |
| A(5Y) | 291.4 | 86.9 | 28.60 | 0.45 | 0.39 | 2.96 | 24.14 | 3.83 |
|  | 291.4 | 86.9 | 28.57 | 0.38 | 0.37 | 2.84 | 23.80 | 4.15 |
|  | 291.4 | 86.9 | 28.46 | 0.45 | 0.42 | 2.89 | 24.76 | 4.20 |
| B(10Y) | 302.7 | 86.8 | 28.32 | 0.48 | 0.38 | 2.92 | 24.24 | 4.07 |
|  | 302.7 | 86.8 | 28.62 | 0.44 | 0.37 | 2.89 | 23.46 | 3.78 |
|  | 302.7 | 86.8 | 28.73 | 0.42 | 0.36 | 2.87 | 23.97 | 4.34 |
| C(15Y) | 276.4 | 75.6 | 28.30 | 0.40 | 0.36 | 2.87 | 24.61 | 3.82 |
|  | 276.4 | 75.6 | 27.92 | 0.41 | 0.36 | 2.91 | 22.44 | 4.03 |
|  | 276.4 | 75.6 | 28.51 | 0.36 | 0.34 | 2.89 | 23.98 | 4.05 |
| D(20Y) | 219.3 | 67.7 | 28.19 | 0.33 | 0.36 | 2.89 | 24.32 | 3.79 |
|  | 219.3 | 67.7 | 28.25 | 0.35 | 0.34 | 2.79 | 22.56 | 4.20 |
|  | 219.3 | 67.7 | 28.18 | 0.32 | 0.37 | 2.88 | 21.56 | 3.82 |
| E(25Y) | 182.5 | 49.1 | 28.18 | 0.31 | 0.34 | 2.85 | 22.07 | 3.70 |
|  | 182.5 | 49.1 | 28.01 | 0.33 | 0.35 | 2.86 | 23.01 | 3.96 |
|  | 182.5 | 49.1 | 28.44 | 0.36 | 0.32 | 2.82 | 21.88 | 4.04 |
